# Water, sanitation and hygiene at sex work venues to support menstrual needs

**DOI:** 10.3389/fpubh.2024.1305601

**Published:** 2024-02-28

**Authors:** Penelope A. Phillips-Howard, Edyth Osire, Cynthia Akinyi, Garazi Zulaika, Fredrick O. Otieno, Supriya D. Mehta

**Affiliations:** ^1^Department of Clinical Sciences, Liverpool School of Tropical Medicine, Liverpool, United Kingdom; ^2^Nyanza Reproductive Health Society, Kisumu, Kenya; ^3^Department of Internal Medicine, Division of Infectious Diseases, Rush Medical College, Chicago, IL, United States; ^4^Division of Epidemiology & Biostatistics, School of Public Health, University of Illinois Chicago, Chicago, IL, United States

**Keywords:** water, sanitation and hygiene (WASH), female sex workers, menstrual health and hygiene (MHH), sexual and reproductive health, reproductive tract infections, waste disposal

## Abstract

**Introduction:**

Adequate menstrual health and hygiene (MHH) is necessary for women's health and equity of all menstruators. Female sex workers (FSW) require good MHH to prevent discomfort and exposure to pathogens. No studies have evaluated water, sanitation, and hygiene (WASH) conditions of FSW. We report on a cross-sectional WASH assessment at FSW venues in Kisumu, western Kenya.

**Methods:**

Stakeholders identified 77 FSW venues in Kisumu, of which 47 were randomly sampled and visited between April–May 2023. A standardized structured survey of WASH conditions was deployed by trained research staff using Android tablets after proprietor's consent. WASH scores ranging 0–3 were computed based on point each for direct observation of water available, soap available, and acceptable latrine. MHH scores ranging between 0–4 were computed (one point each) for direct observation of: currently available soap and water, locking door on a usable latrine, functional lighting, and a private area for changing clothes or menstrual materials, separate from the latrine(s). WASH and MHH scores were compared by venue type using non-parametric Kruskal-Wallis tests, and non-parametric Spearman rank tests.

**Results:**

Full WASH criteria was met by 29.8% of venues; 34.0% had no adequate WASH facilities; 46.8% had no female latrine; and 25.5% provided soap and water in private spaces for women. While 76.6% had menstrual waste disposal only 14 (29.8%) had covered bins. One in 10 venues provided adequate MHM facilities. Poorest WASH facilities were in brothels and in bars, and three-quarters of bars with accommodation had no MHH facilities.

**Discussion:**

WASH and MHH services were sub-optimal in the majority of FSW venues, preventing menstrual management safely, effectively, with dignity and privacy. This study highlights the unmet need for MHH support for this population. Poor MHH can deleteriously impact FSW health and wellbeing and compound the stigma and shame associated with their work and ability to stay clean. Acceptable and cost-effective solutions to sustainably improve WASH facilities for these populations are needed.

**Trial registration:**

Clinicaltrial.gov NCT0566678.

## 1 Introduction

Urbanization is projected to reach 62% of the population across sub-Saharan Africa by 2050 (1). Interventions have been initiated to address urban stress, deprivation, and structural inequities but most face contextual, socio-political, institutional, and resource challenges (2). Inadequacies in water, sanitation and hygiene (WASH) facilities in living and working environments and related exposure to health hazards is a major consideration (3). Sustainable Development Goal 6 recognizes safe WASH as a basic human right necessary to ensure health and wellbeing (4). In March 2023, the World Health Organization (WHO) and the United Nations Children's Fund called on all nations to radically accelerate action to make WASH a reality for all (3).

Adequate WASH is required for all menstruators to urinate, defecate and manage their menstruation with privacy, safety, and comfort (4), and is a basic rights of all menstruators, essential for girls' dignity, and key to ensuring equity in health and education (5). In 2012, the WHO Joint Monitoring Programme affirmed the need for reliable access to water and soap for body and handwashing, sanitation options that are clean and private, sustainable disposal amenities, and access to clean menstrual hygiene materials, both at home and away from home (6). More recently menstrual health and hygiene (MHH) has become more prominent, encapsulating the physical and psychological aspects of menstruation, and broader systemic factors that link menstruation with health, wellbeing, gender equality, education, equity, empowerment, and human rights (7).

Studies on the menstrual and WASH needs of economically vulnerable persons, who rely on sex for livelihood such as female sex workers (FSW), are largely related to evaluations of intravaginal practices in relation to HIV and STI risk or potential use of vaginal rings. Such evaluations illustrate the need for soap and water for hygiene across cultures (8); for example, in Tanzania, water, water and soap, or other agents were reported as essential for cleaning vaginal secretions (“dirt”), menstrual blood and post-coital discharge (9). Sex workers in Cambodia reported stigma, and discrimination associated with their ability to clean themselves intravaginally (10). Caruso and colleagues recommend a gender-based goal for WASH is necessary to understand how compromised resources affect women, noting for example, that menstrual hygiene needs often are not considered in design and delivery of WASH, with implications for satisfaction and safety (11).

Sanitation insecurity poses a threat to women's safety, wellbeing and dignity (12). Psychosocial stress was found to be a common response to inadequate WASH access among females in international research (13). In Kenya, lack of sanitation has been shown to increase non-partner violence (14), and causes shame and marginalization (15). In rural Kenya, poor WASH and subsequent menstrual practices in adolescent girls and young women have been associated with bacterial vaginosis (16, 17). WASH-related challenges are common in Kisumu County in western Kenya where our study takes place: while 71% of the population obtains water from an improved water source, 17% rely on surface water; 26% use unimproved sanitation services, with an additional 4.8% resorting to open defecation (18). In a 2017 national household survey of 4,556 Kenyan women aged 15 to 49 who menstruated in the past 3 months, 80% reported disposing of menstrual materials in the toilet/latrine and 22.5% reported using sleeping area as their main location for MHH (19).

In a nation-wide key population size estimation exercise conducted 2017–2018 by the National AIDS and STD Control Program (NASCOP) of the Kenyan Ministry of Health (20), estimates of FSWs ranged from 129,271 to 206,609, with the mean being 167,940. Improving WASH facilities and resources to support MHH in the workplace is essential for health, wellbeing, and productivity, though to our knowledge, venues where sex work occurs have not been specifically called out as workplaces in relation to WASH and MHH studies (21). This article presents data on WASH facilities at venues where sex work takes place in Kisumu, Kenya, to determine whether any deficits identified may impact participants' ability to manage their menstrual needs. These aspects could be important confounders or mediators to any MHH intervention and could directly affect individual hygiene behaviors.

## 2 Methods

### 2.1 Study site

This study was conducted in Kisumu, western Kenya. Located on the shores of Lake Victoria, Kisumu is approximately ~320 km from Nairobi. Kisumu houses a population of ~800,000 persons, extending to 1.2 m when including the wider metropolitan area. The population is of mixed heritage, but predominantly of Luo ethnicity. Close to half (47%) of residents live in informal settlements including Kondele, Obunga, Nyalenda, Nyawita, and Manyatta (22). Similar to health, oversight of WASH facilities are devolved to the county Ministry of Health, through their WASH Division in Kisumu. However, in a vulnerability mapping exercise carried out by UN-Habitat, in seven informal settlements nearly three-quarters (74%) of water points were managed by individuals (e.g., business owners), with the majority charging for access, and only 5% publicly managed by county or national government (23).

### 2.2 Study design and sampling

This WASH study is a sub-study to a parent study designed to evaluate the effectiveness and safety of menstrual cups for FSW. Briefly, the study is a single arm trial which began February 8, 2023 (ClinicalTrials.gov NCT05666778; Pan African Clinical Trials Registration PACTR202305912778108), in which FSW undergo a control phase of 1 year of observation of MHH and sexual practices and incidence of Bacterial vaginosis (BV) and sexually transmitted infections (STIs). After this control phase, they are provided with a reusable menstrual cup that can be worn during sex, followed by another year of follow-up for MHH and sexual practices, BV, and STIs. While menstrual cups have a good safety profile (24), our parent study is assessing cup contamination, and WASH factors may affect this.

#### 2.2.1 Selection of sex work venues for WASH assessment

For the WASH assessments in this cross-sectional study, we first identified hot spots, as places where FSW meet or congregate, meet one another to socialize, and also solicit or have sex with their clients. The hotspots are referred to as venues, and include brothels, bars, restaurants, and guesthouses. The initial designation of brothel, bar, restaurant, or guesthouse was determined by FSWs at the time of initial hotspot listing, and was confirmed or revised upon in-person visit by the study team. Descriptions of venues are provided in Box 1, and follow the typologies used in national FSW hotspot mapping (20). Some venues can be a single-typology enterprise, while others can be combination typology, for example where alcohol is sold, has a lodging, and serves food.

Box 1 **Description of sex work venue types assessed for WASH in Kisumu, Kenya, 2023.**

**Sex Den/Brothel:** These are premises explicitly dedicated to providing sex. They are more secure than the streets. The brothels are rented by the female sex workers, paid for on an hourly or daily basis. Typically, in the venues assessed in this study, brothel rooms ranged from 100 KSH to 500 per day KSH, and could be charged per night or per number of hours.

**Street/Highway/Alley:** These are streets, alleys and highways where sex workers solicit for sex during the day, at night or late evening. They may have sex in vehicles, short stay lodges or on the streets.

**Restaurant:** An enterprise that prepares food and serves to customers. Restaurants may also serve beverages containing alcohol.

**Bar**: A bar is an alcohol vending venue. Clients that go to this venue are met by a sex worker and solicitation happens at the bar, after which, depending if lodges are available within the bar, they can proceed to have sex there. There are bars without lodging, and this means that the client and the sex worker go elsewhere. Some bars also have restaurants within the venue. Some sex workers have sex in bars without lodging, in the areas with poor lighting and designated for sex. This is not with authority from the owners, but it happens.

**Guest house**: This is a type of lodging that is less expensive and less formal than a hotel. Sex workers who solicit for sex on the street or bars without lodging may have sex with clients in the more affordable guest houses. Typically, in the venues assessed in this study, guest house rooms ranged from 300 to 1,500 KSH per room per night, with hours of occupancy allowed generally from 8 or 9 p.m. to between 7 to 8 a.m.

#### 2.2.2 Sampling venues

Peer leaders from two community organizations that support FSW in Kisumu, Keeping Alive Society's Hope (KASH) and Kisumu Sex Workers Alliance (KISWA), identified and engaged venues where sex work takes place. The peer leaders were current or former FSW. These peers are registered with KASH and KISWA and are the focal persons for FSW in their specific hotspot. They are key in educating FSW on issues of health (including HIV testing and PrEP counseling), safety, and managing clients and daily work. The peers come from different regions within Kisumu Central Subcounty. Each peer leader had her own area of town with minimal overlap to another person's area. From an initial listing of 80 hotspots, there were 3 redundancies which were removed. From the list of 77 hotspots, a 50% simple random sample was generated (*N* = 39), stratified on venue type (Table 1) and area of town. Street based locations and venues located in the central business district were under-represented, and a second sample was drawn to increase central business district representation, for a total of 47 venues to be assessed.

**Table 1:** Distribution of characteristics at 47 sex work venues in Kisumu, Kenya.

	***N* = 47**
	***n*** **(%)**
Venue type	
Street-based	3 (6.4)
Guest House/lodging	5 (10.6)
Bar	15 (31.9)
Brothel	6 (12.8)
Guest House/lodging + Bar	4 (8.5)
Restaurant + Bar	6 (12.8)
Guest House/lodging + Restaurant + Bar	8 (17.0)
Latrine types and availability	
Type of toilets/latrines	
Flush/pour-flush to sewer	35 (74.5)
Flush/Pour-flush to tank or pit	1 (2.1)
Pit latrine with slab/covered	10 (21.3)
Bucket	1 (2.1)
Separate latrines for women and men	
Yes, completely separate latrines	32 (68.1)
Latrines are joined and there is a privacy wall	1 (2.1)
Common use latrines only	14 (29.8)
Total number of toilets available to women only	
0	23 (48.9)
1	14 (29.8)
2	5 (10.6)
3–4	6 (10.6)
Mean proportion of toilets available to women that are usable	93.8%
Total number of toilets available to men only	
0	16 (34.0)
1	10 (21.3)
2	14 (29.8)
3–4	7 (14.9)
Mean proportion of toilets available to men that are usable	100%
Total number of common use toilets available	
0	18 (38.3)
1	11 (23.4)
2	8 (17.0)
3–4	4 (8.5)
5–12	6 (12.8)
Mean proportion of common use toilets that are usable	94.8%
Total number of latrines counted on premises	183
Total number of latrines assessed	151 (82.5%)
Proportion of latrines with…	
Unstable flooring	11.1%
Holes in wall	12.9%
Strong offensive smell	51.0%
Urine or feces on the floor	42.6%
No roof	3.5%
No door	24.3%
No locking door	65.8%
No functional lighting	37.3%
There is at least one usable toilet/latrine accessible to women only, at all times	22 (46.8)
Where are the women's toilets located?	
Within building	25 (53.2)
Outside building but on premises	13 (27.7)
Off premises (for all 7, distance was reported as < 1 min walk)	7 (14.9)
Some within building, and some outside building but on premises	2 (4.3)
How clean are the toilets	
Not clean	9 (20.0)
Somewhat clean	18 (40.0)
Clean	18 (40.0)
Are latrines or septic tanks emptied (or latrines are safely covered when they fill)	
No	36 (76.6)
Yes	10 (21.3)
Unknown	1 (2.1)
How is solid waste (garbage) from the venue disposed of	
Private waste collection system	44 (93.6)
Burned on premises	1 (2.1)
Openly dumped on premises	2 (4.3)
Water availability	
Main source of drinking water	
Piped	29 (61.7)
Protected well	1 (2.1)
No water source	2 (4.3)
Purchased in Jerry cans	15 (31.9)
The venue treats the water from the main source to make it safe to drink	
No	37 (82.2)
Yes (3 piped source, 1 protected well, 4 Jerry cans)	8 (17.8)
Boiling	2
Chlorination	6
No water source (excluded from percent calculations)	2
Drinking water is currently available	29 (61.7)
Piped	21 (72.4)
Protected well	0 (0)
Jerry cans	8 (53.3)
No water source	2
Number of drinking water taps at the venue	
0^*^	23 (48.9)
1	21 (44.7)
2	2 (4.3)
12	1 (2.1)
There are handwashing facilities at the venue	30 (63.8)
WASH assessment	
Water is currently available	28 (59.6)
Soap is currently available	22 (46.8)
There is at least one acceptable latrine	20 (42.6)
WASH Score (sum of one point each for water, soap, latrine)	
0	16 (35.6)
1	6 (13.3)
2	9 (20.0)
3	14 (31.1)
Conditions for menstrual hygiene and health
For venues with handwashing facilities, current availability of soap and water	
Neither water nor soap	2 (6.7)
Water only	8 (26.7)
Water and soap	20 (66.6)
Are water and soap available in a private space for women to manage menstrual hygiene?	
No, no water or soap in a private space	32 (68.1)
Yes, water but no soap	3 (6.4)
Yes, water and soap	12 (25.5)
Separate from the toilets/latrines, there is a private place where women can change their menstrual materials	10 (21.3)
There are disposal mechanisms for menstrual hygiene waste at the venue	36 (76.6)
How are sanitary pads disposed of at the venue	
Thrown in pit latrine	6 (12.8)
Placed in a regular trash bin and collected by municipal services	36 (76.6)
Women take them with them	5 (10.6)
There are covered bins for disposal of menstrual hygiene materials	14 (29.8)
MHH Score (one point each for: latrine with locking door, latrine with functional lighting, soap and water currently available and located at the toilet, a private place separate from toilets/latrines)	
0	14 (31.1)
1	9 (20.0)
2	14 (31.1)
3	4 (8.9)
4	4 (8.9)

^*^Of 23 venues with no drinking water tap: 2 are from the venues with no water source, 10 are at venues that rely on water purchased in Jerry cans, 11 are from sources with piped water. Of the 11 with piped water, 8 did not have water currently available. Of the 3 with currently available piped water, they were classified as “no drinking water taps” because the pipes are inside the premises and not publicly available.

#### 2.2.3 Venue recruitment and consenting

The peer leaders were informed of the 47 identified venues and the proposed dates for the visits. They then visited the venues to inform the proprietors of the request to visit, the purpose of visit (to do a WASH assessment for a research study), and the desired dates of the visits. They then communicated back to the Project Coordinator (EO) to confirm acceptance by the proprietors. All the visits were arranged to take place on weekdays and during daytime hours (9 a.m.−4 p.m.). Visits were arranged in advance. Upon arrival at the venue, the study explanation and consent form were read to proprietors in their preferred language (Kiswahili, DhoLuo, or English) and signed before the WASH assessment was done. No personal or identifying information was collected from the proprietor. Conditions for the three street venues were assessed for the nearest location at which FSW had an agreement that they could use the facilities, though sex work (solicitation, sex acts) was not reported to take place at these locations. One was a restaurant (without bar) and the latrine was within the building; the other two were bars with lodging and the latrines were within the premises.

### 2.3 Data collection

Researchers trained on WASH measurement made assessments using a standardized coded survey questionnaire, adapted from the United Nations High Commissioner for Refugees WASH in schools checklist (25). The following measures were assessed (Supplementary material for full survey): access to a latrine with inside locking door, clean water, and soap; primary source of and distance to nearest water (pump, borehole, piped, etc.); conditions of latrine (stability of floor/platform, holes in wall, offensive smell, feces or urine pooled on floor). Locations having a private place for FSW to wash or change MHH were recorded as separate features. Bathroom facilities are referred to as a latrine throughout this report and may or may not have included a toilet or other fixed receptacle for collecting urine or feces. Researchers were trained on WASH assessments, with two (EO, CA) attending each assessment, with additional researchers supporting them. One researcher entered findings directly into an electronic Android tablet hosting Open Data Kit (ODK), while one entered finding on a paper form. Disagreements and discrepancies were discussed in real time while at the venue; if not resolved onsite, they were discussed subsequently with the PI (SDM) and technical officer (GZ). Not all women or females menstruate, and not all menstruators are women or females; we use the term women and FSW throughout this report following the WASH checklist wording (25) and the study's target population.

### 2.4 Data processing of WASH and MHH scores

#### 2.4.1 WASH score

In order to compare across locations, we generated WASH scores ranging 0–3 modified from Alexander et al. (26). The score was comprised of one point each assigned for direct observation of: water available, soap available, and acceptable latrine. “Acceptable” latrines were defined as having all of the following features: clean (no visible feces on floor), no strong/offensive smell, having door and roof, no major holes in walls, stable floor or platform (26). Venues were classified as having an acceptable latrine if there was at least one latrine meeting these criteria. Latrines were also categorized as to whether or not they were usable (defined as available, functional, and with a closable door that locks) (25), and a latrine could be usable, while not being acceptable. For example, an unclean latrine with strong/offensive smell and non-functional lighting could meet the definition for usable, though not acceptable.

#### 2.4.2 MHH score

A MHH score ranging between 0–4 was determined based on one point each for: currently available soap and water, locking door on a usable latrine, functional lighting, and a private area for changing clothes or menstrual materials, separate from the latrine(s). The score aspects of presence of soap and water, locking door on usable latrine, and private area were adapted from adapted from Alexander et al. (26), with “locking door on a usable latrine” substituting for a “privacy wall” as we felt that insufficient in these settings. Functional lighting was included in the MHH score, given that a large portion of sex work takes place after dark, and given the types of work setting (brothels, bars, street-based). Inclusion of a private area, functional lighting, and lockable door is supported by prior research indicating these as user priorities among residents of low-income urban settlements, including in Kisumu (27). All latrines had to have functional lighting and all doors had to be locking to be assigned a point for each, respectively.

### 2.5 Statistical analysis

Characteristics of sex work venues are represented with frequency distributions. WASH and MHH scores were compared by venue type using non-parametric Kruskal-Wallis test. The correlation between WASH and MHH score was done using non-parametric Spearman rank test. Statistical analyses were conducted using Stata/SE v17. For two venues (one bar, one brothel), WASH and MHH scores could not be calculated because they each had one latrine that was reported to be in use at the time of the assessment. These two locations did have soap and water available on premises, and locking doors, but acceptability of the latrine could not be assessed, and they did not have private areas separate from latrines for changing menstrual materials.

## 3 Results

### 3.1 WASH facility settings

WASH assessments were conducted between 18th April to 5th May 2023, with one conducted earlier in February to test the tools. In total, 47 venues were contacted, all 47 proprietors consented to WASH assessment, and WASH facilities at 47 venues used by FSW were observed (Table 1). Over a third (38.3%) of venues were bars offering food or accommodation, or both; one third (32%) of venues were bars only, with the remaining venues representing brothels (12.8%), accommodation (10.6%), and on the street (6.4%). Garbage was predominantly (93.6%) disposed through private waste collectors.

### 3.2 WASH evaluation

**Figure 1:**
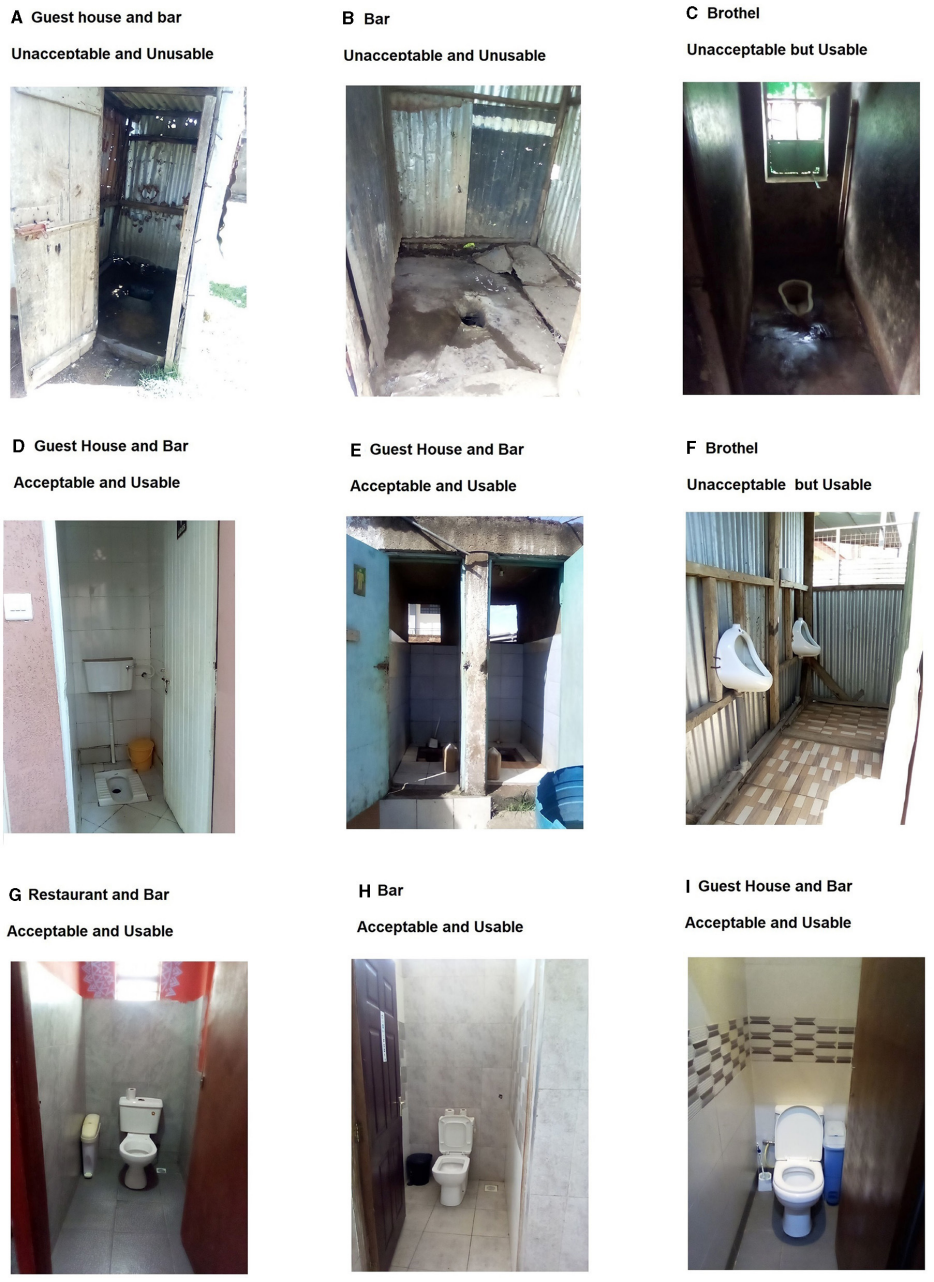
Examples of latrines and classification of acceptability and usability. **(A)** This latrine from a Guest House and Bar was classified as unacceptable and unusable: the flooring was unstable, there were holes in the walls, there was no functional lighting, and it was very unclean with strong smell of urine and feces. Notably, this latrine was also shared with the community tenants. **(B)** This latrine from a Bar was classified as unacceptable and unusable: the floor was very unstable (“can sink at anytime” was noted by investigators doing the assessment), the door had no lock, and it was very unclean and had a strong smell of urine and feces. **(C)** This latrine from a Brothel was classified as unacceptable but usable: It was very unclean with urine on the floor, pungent smell of urine and faces, and no functional lighting. **(D)** This latrine (labeled as “Ladies”) from a Guest House and Bar was classified as usable and acceptable: being somewhat clean, no strong odors of urine or feces, locking door, stable flooring, no holes in wall, and functional lighting. Investigators also noted a covered bin for disposal of MHH materials. At this venue, water was available, but soap was not. **(E)** This latrine from a Guest House, Restaurant, and Bar was classified as acceptable and usable: a wall separated male and female toilets, flooring was stable, doors were lockable, there was functional lighting and no strong smell of urine or feces. A handwashing point was located outside the latrines, and water was available but not soap. This venue had multiple handwashing points and the one near the restaurant had water and soap available. Additionally, there were handwashing points near or inside the guest rooms with both water and soap available. **(F)** This latrine at a Brothel is usable but unacceptable. It was clean with no smell of urine, and stable flooring, but with no door, no functional lighting, and limited privacy. While not directly applicable to females, we provide this example of a urinal as it was representative of several urinals observed. **(G)** This latrine from a Restaurant and Bar was classified as acceptable and usable: very clean, with locking door, stable flooring, functional lighting, and a covered bin for disposal of used menstrual materials. There were multiple handwashing points with water and soap. **(H)** The latrine at this Bar was classified as usable and acceptable: very clean, with functional lighting, locking door, stable flooring, a covered bin for disposal of used menstrual materials, and handwashing facilities with water, soap, and a towels for drying hands. **(I)** The latrine at this Bar was classified as usable and acceptable: very clean, with functional lighting, locking door, stable flooring, a covered bin for disposal of menstrual materials, and handwashing facilities with water, soap, and a functional, air-blowing hand dryer available. The photographs were taken using the study tablets via the ODK programming, with express permission of the venue proprietors who consented to the WASH assessment.

Nearly one third (29.8%) of venues were observed to have water, soap and usable latrines, and just over one third (34.0%) had no observed adequate WASH facilities (Table 1). The majority (74.5%) of latrines were flush linking to the municipal sewer system, or pit latrines (21.3%). Two thirds of latrines (68.1%) were separate for women and men. Latrines were frequently of poor quality, with two-thirds having no locking door, half with a strong offensive smell, and close to half with urine or feces on the floor (Figures 1A–C). A quarter had no door and a third no lighting. Close to half (46.8%) had no female latrine, with 14 facilities having one latrine and 11 with two or more latrines. Half (53.2%) of latrines were located within the venue and a further quarter on the premises but outside the main building. In these same facilities, one third had no latrine for males, 10 had one and 21 had two or more male latrines. Two-thirds of premises had unisex latrines, with 10 locations having three or more latrines. Over nine out of every ten latrines were observed to be usable, with one in five not clean. Ten venues reported that they emptied latrines or septic tanks when full. Overall, just under half (46.8%) of facilities were observed to have at least one usable latrine accessible to women. Less than one-third of venues met full WASH criteria, and those with the highest standards are exemplified in Figures 1G–I. Those considered to be “average” by study staff are depicted in Figures 1D–F. It should be noted that while investigators classified some latrines as unusable (Table 1, Figures 1A, B), these latrines were all technically being used by the venues.

### 3.3 Water source and availability

Two-thirds (61.7%) of venues had piped drinking water, and an additional third of venues obtained water through purchasing in Jerry cans, with two-thirds of venues having water available for drinking at the time of survey. Nearly half (49%) of venues had no observed water drinking taps: 2 at venues with no water source, 10 at venues in which water was purchased in Jerry cans, and 11 from sources with piped water. Of these 11 venues with piped water, 8 did not have water currently available, and for 3 venues water was currently available, though taps were not available as they were inside the premises (e.g., a kitchen or private room) and could not be accessed by patrons. Four out of five venues did not treat water to ensure it was safe for drinking. Two-thirds (63.8%) had handwash facilities, with ~60% having water and under half (46.8%) having soap.

### 3.4 WASH for MHH

While two thirds of venues had water and soap available, only one quarter (25.5%) were observed to provide this in private spaces for women, and a private place to change was available in one in five venues. Three quarters (77%) of venues could dispose of MHH waste; this was predominantly through bins with waste then collected by municipal services with general trash (i.e., no venue reported separate collection services for MHH waste). However covered bins were observed in less than a third (29.8%) of venues. Less than one in ten venues (8.9%) fulfilled the criteria of providing adequate MHH services, in terms of a latrine with locked door, functional lighting, soap and water located at the latrine, with a private place to change. Nearly one third (31%) of venues fulfilled none of these criteria.

### 3.5 Comparison of venue types and WASH facilities

**Figure 2:**
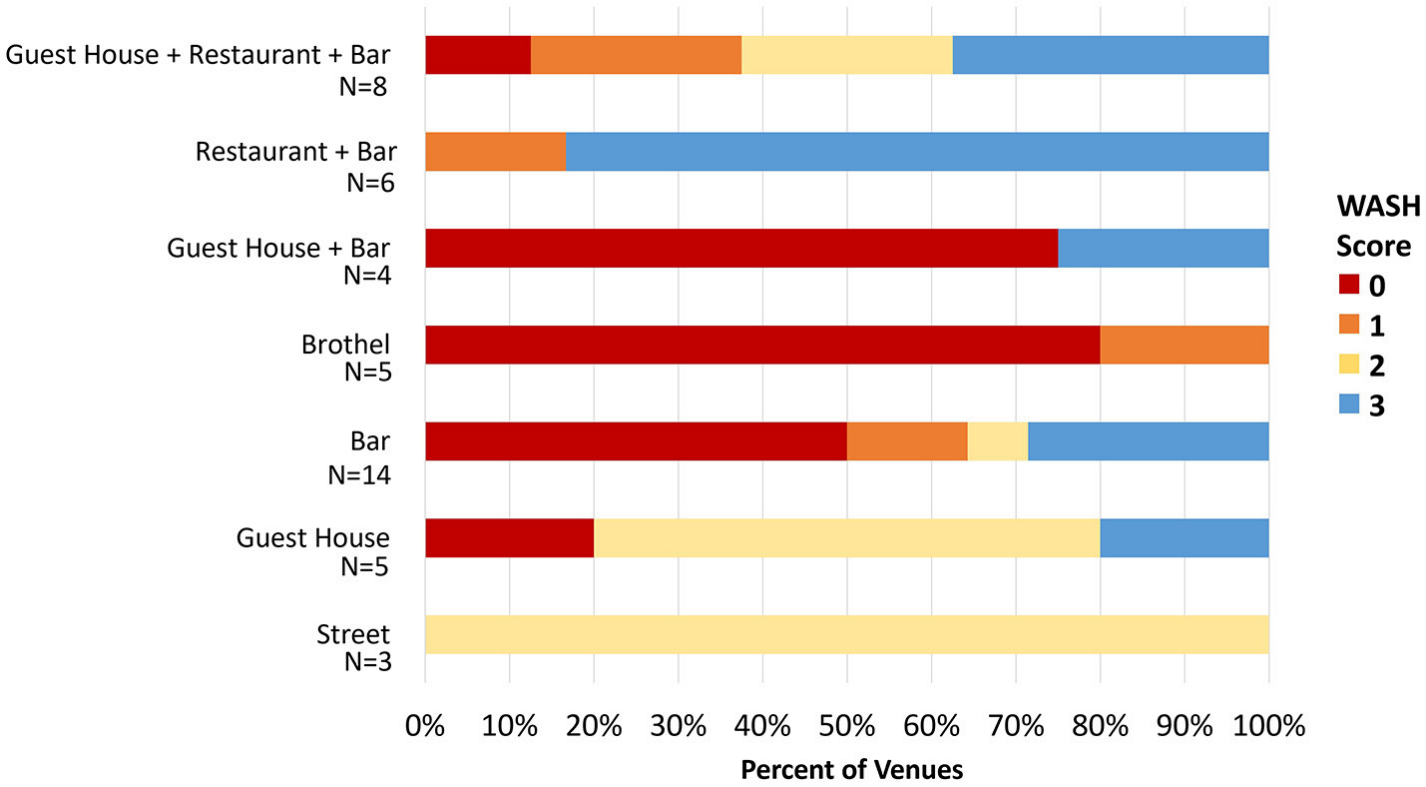
Distribution of WASH score by venue type. The X-axis shows the percentage of venues meeting a particular WASH score, which is represented by the legend to the right. For example, for venues that are classified as Guest House and Bar (*N* = 4), 75% had a WASH score of zero and 25% had a WASH score of three.

The WASH score distribution differed by venue type (*p* = 0.044, Kruskal-Wallis test) (Table 2; Figure 2). Across venues, the poorest WASH availability was in brothels, bars alone, or bars with accommodation, with 75.0%, 66.7%, and 46.7% respectively having no WASH facilities available (Table 2). The highest mean and median scores for WASH facilities were in venues classed as bar with restaurants, the majority having water, soap, and at least one acceptable latrine, while brothels and bars with accommodation had the lowest scores with few facilities available for women.

**Table 2:** Distribution of availability of water, soap, acceptable latrine and WASH score by venue type.

**Venue type**	**Nothing available (i.e., WASH score = 0) *n* (%)**	**Water available *n* (%)**	**Soap available *n* (%)**	**At least one acceptable latrine available for use of women *n* (%)**	**WASH score mean/median (range)**
Street^*^, *N =* 3	0 (0)	3 (100)	2 (66.7)	1 (33.3)	2/2 (2–2)
Guest house, *N =* 5	1 (20)	4 (80)	3 (60)	2 (40)	1.8/2 (0–3)
Bar, *N =* 14	7 (50)	7 (50)	4 (28.6)	5 (35.7)	1.1/0.5 (0–3)
Brothel, *N =* 5	4 (80)	1 (20)	0 (0)	0 (0)	0.2/0 (0–1)
Guest house and bar, *N =* 4	3 (75)	1 (25)	1 (25)	1 (25.0)	0.8/0 (0–3)
Restaurant and bar, *N =* 6	0 (0)	5 (83.3)	5 (83.3)	6 (100)	2.7/3 (1–3)
Guest house and restaurant and bar, *N =* 8	1 (12.5)	5 (62.5)	5 (62.5)	5 (62.5)	1.9/2 (0–3)
Total (% of 45)	16 (35.6%)	26 (57.8%)	20 (44.4%)	20 (44.4%)	1.5/2 (0–3)

^*^WASH conditions for street based sex work were assessed for the nearest location at which FSW had an agreement that they could use the facilities. These were (one was restaurant without bar and latrine was within building, the other two were bars with lodges and the latrines were within the premises), though sex work (solicitation, sex acts) was not reported to take place at these locations.

### 3.6 Comparison of venue types and MHH facilities

**Figure 3:**
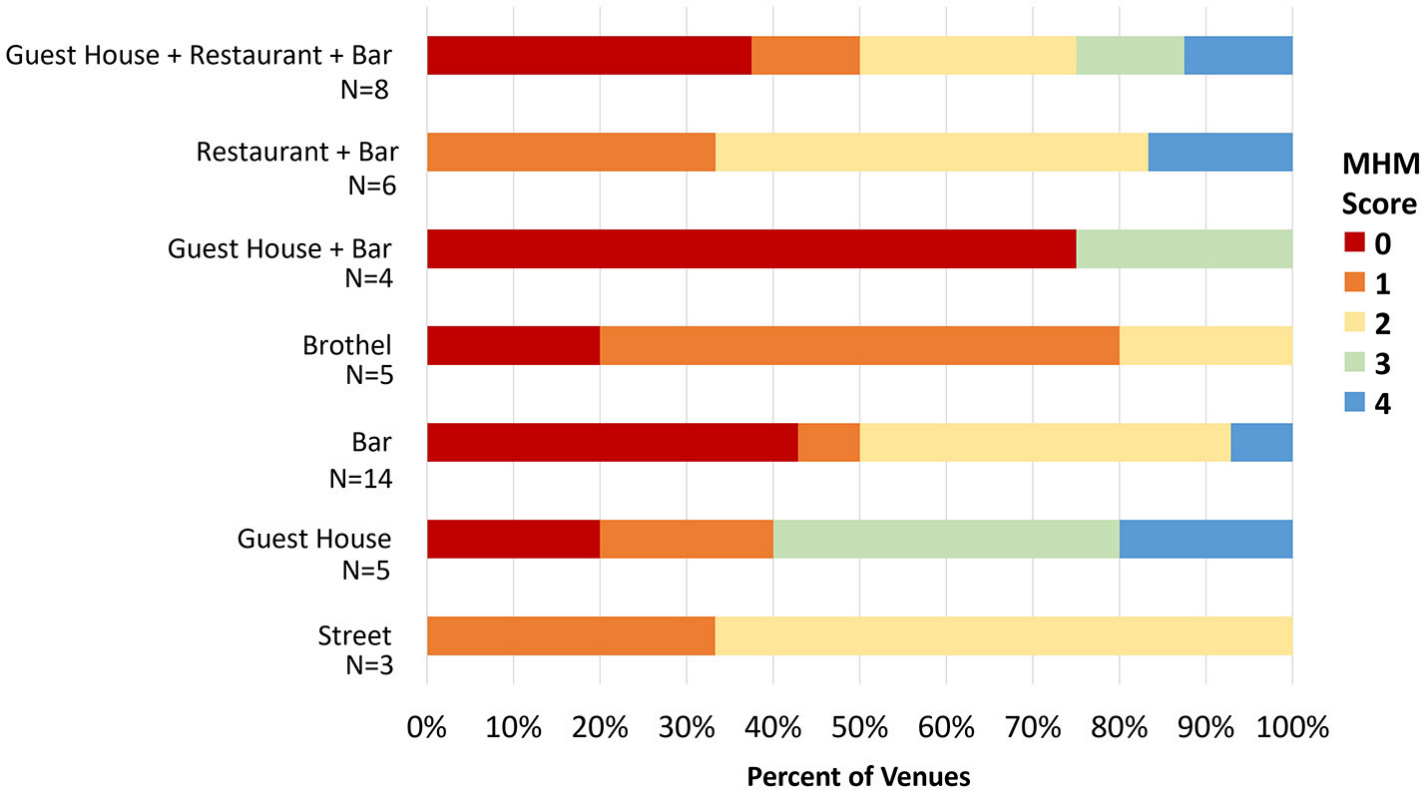
Distribution of MHH score by venue type. The X-axis shows the percentage of venues meeting a particular MHH score, which is represented by the legend to the right. For example, for venues that are classified as Bar (*N* = 14), 43% had a MHH score of zero and 7% had a MHH score of one, 43% had a MHH score of 2, and 7% had a MHH score of 4.

**Figure 4:**
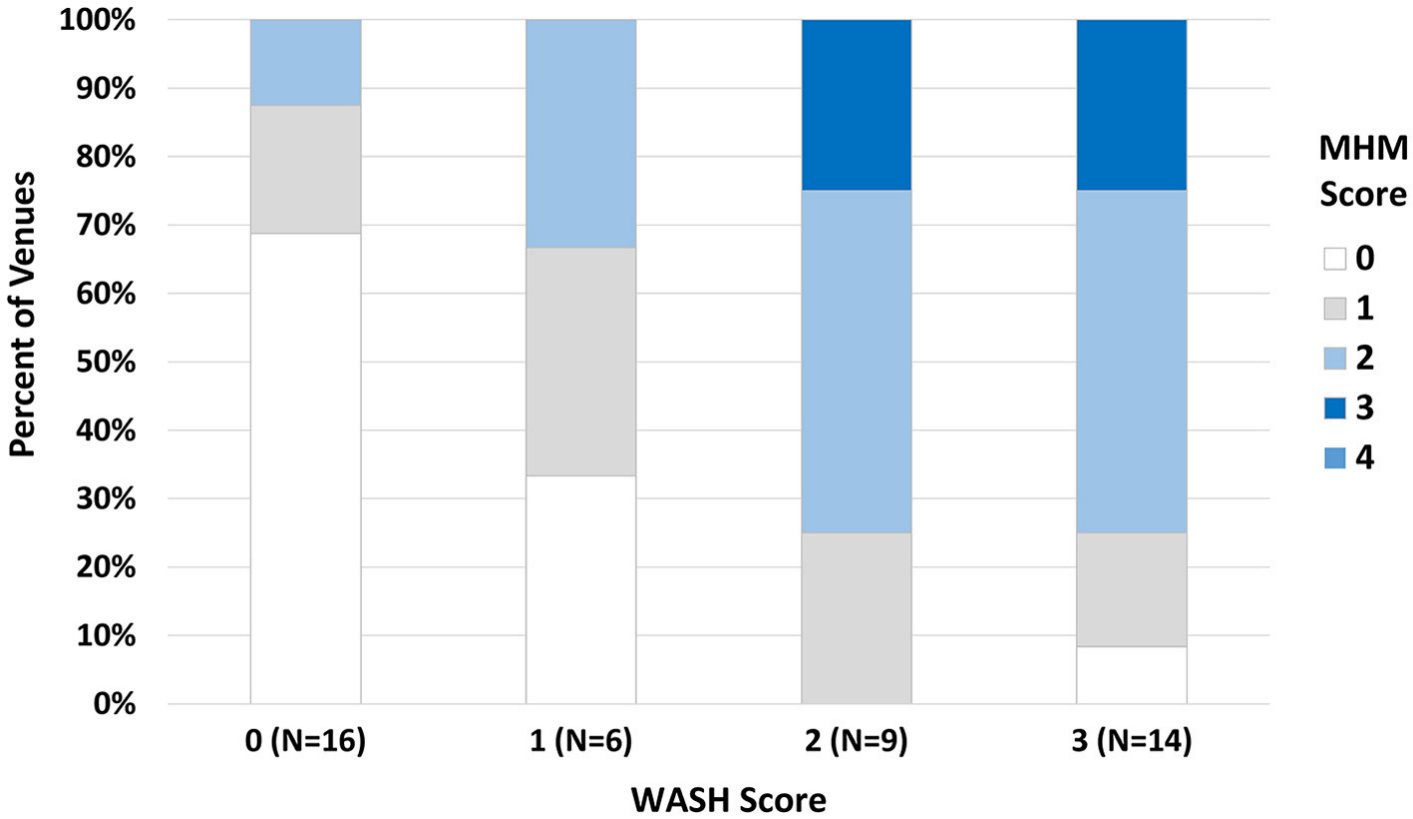
Distribution of MHH score by WASH score. The y-axis shows the percent of venues having an MHH score ranging 0–4 (legend, right), stratified by WASH score ranging 0–3, as depicted on the x-axis. For example, among 16 venues with a WASH score of zero, nearly 70% of those also had a MHH score of zero.

Overall, there was no statistically significant difference in MHH score by venue type. Only 4 (8.9%) venues achieved an MHH score of 4, while 31.1% had a score of zero (Table 3; Figure 3). The highest proportion of venues classed as bars with accommodation, or bars alone had no MHH support, e.g., with 75% and 42.9% scoring zero on the MHH score (Table 3). WASH score and MHH score were positively correlated (Spearman's rho = 0.66, *p* < 0.001) due to some related elements (e.g., doors and locking door being components of “acceptable” latrine within WASH score) (Figure 4).

**Table 3:** Distribution of menstrual health and hygiene score and score components by venue type.

**Venue type**	**Nothing available (i.e., MHH score = 0) *n* (%)**	**Water and soap available at latrine *n* (%)**	**Latrine with door that locks *n* (%)**	**Functional lighting at latrine *n* (%)**	**Private space separate from latrines where women can change pads or clothes *n* (%)**	**MHH score** **mean/median (Range)**
Street based, *N =* 3	0 (0)	3 (100)	1 (33.3)	2 (66.7)	0 (0)	1.7/2 (1–2)
Guest house, *N =* 5	1 (20)	1 (20)	2 (40)	4 (80)	3 (60)	2.2/3 (0–4)
Bar, *N =* 14	6 (42.9)	7 (50)	3 (21.4)	8 (57.1)	1 (7.1)	1.2/1.5 (0–4)
Brothel, *N =* 5	1 (20)	1 (20)	2 (40)	2 (40)	1 (20)	1/1 (0–2)
Guest house and bar, *N =* 4	3 (75)	0 (0)	1 (25)	1 (25)	1 (25)	0.75/0 (0–3)
Restaurant and bar, *N =* 6	0 (0)	5 (83.3)	0 (0)	3 (50)	2 (33.3)	2/2 (1–4)
Guest house and restaurant and bar, *N =* 8	3 (37.5)	5 (62.5)	2 (25)	3 (37.5)	2 (25)	1.5/1.5 (0–4)

## 4 Discussion

WASH facilities were found to be sub-optimal in nearly all sex work venues assessed, with only three (7%) observed to have adequate MHH and WASH (scores). While in most instances FSW could find somewhere to urinate, defecate, and manage their menstruation, the environments were often unacceptable and lacked the components needed for them to manage their menstrual needs safely, effectively, with dignity and privacy. One third of the facilities had nothing available for WASH.

Access to safe WASH is a well-recognized human right and is vital for health and wellbeing (4). This encompasses health for all (3), regardless of type of employment and social status (28). A strong evidence base has been built on the WASH needs of school-going girls (29–31), and on WASH facilities for women, with exemplar studies in India focusing on sanitation insecurity (12), and research illustrating associations between poor WASH and urogenital infections (32, 33), and women's mental health (34). We found no evidence of any studies examining access or quality of WASH facilities for menstruating FSW in the literature, other than the vaginal ring studies noting that FSW need access to soap and water to clean secretions, menstrual blood and post-coital discharge (8, 9). Anecdotal reports from brothels in Bangladesh and sex workers in India also observe lack of access to facilities with adequate WASH and MHH standards, and link this to issues of human rights and dignity (35, 36). Studies note that FSW encounter stigma and discrimination if they are unable to clean themselves intravaginally (10). Other studies among community members (agree) that poor WASH decreases wellbeing, with one study in western Kenya noting anxiety, frustration, embarrassment, negative identity, marginalization and a lack of self-efficacy (15).

In our study, a lack of WASH facilities was most notable in premises solely providing accommodation, with somewhat improved facilities if a bar or restaurant was attached. The reason that WASH and MHH scores were better at bars or places with a restaurant is that they need a permit to do business and fall under national regulations. The Kenyan National Food Safety Regulations mandate food serving outlets to maintain good hygiene, with a legal requirement to provide sanitation and handwashing facilities (37). Owners are thus held accountable. Studies on accountability for WASH conditions in childcare centers have reflected on the importance of social accountability in informal settlements in Nairobi (38). Poor WASH in establishments frequented by FSW that are not restaurants or bars appear to have few mandatory regulations, thus limiting accountability for WASH and MHH standards. In Nairobi, in informal settlements, a strong association was found between absent landlords and poor WASH facilities (22).

During conversations with proprietors, they often noted the cost of private disposal services or of purchasing water and soap as barriers to providing adequate services. Because the benefits of WASH interventions may not accrue directly to these venues, novel programs that integrate financial incentives or subsidies, improved technology, and positive behavior change interventions may be necessary to raise standards for improved latrines, clean water access, and appropriate waste disposal at venues where FSW engage in sex work (39). At some venues, some proprietors were unaware of the need for covered disposal or of having handwashing facilities and disposal bins near the latrines. While structured research is needed on the barriers, facilitators, costs, and potential benefits of improving WASH and MHH standards in these settings, initial steps may include education that incurs minimal costs (e.g., the need for covers for bins, to place them near latrines, to place handwashing facilities near latrines).

We note that at 43% of venues, latrines were outside the premises. The study team conducting the WASH assessments, comprised of female Project Coordinator (EO) and Study Nurse (CA) who were accompanied by female peer leaders and accompanied in 25% of assessments by female PI (SDM) or female technical officer (GZ), observed that this would require going off premises to remove the MHH material and conduct any personal washing, and then after the sexual encounter, returning to the off-site latrine to clean themselves if the place of sex was not private and did not have water or soap. Distant WASH facilities, with no safe changing space, greatly complicate this common approach and potentially can create harm, e.g. if there is no privacy, or lighting, and women changing can be taken against their will and violated both physically and sexually (14). Moreover, we noted the disposal was largely inadequate across all venue types. Commonly, MHH materials were included in mixed trash.

Menstruation is recognized to be of public health importance. WASH in schools has been prioritized, and the Kenyan government has built a cadre of experts who, in consultation with cross-sectoral expertise, has developed and published strategies on MHH, policy guidelines, and a training handbook for teachers (40–42). The strategy document and training guide note the variety of products available for menstrual care, however, in practice, only disposable sanitary pads are offered as part of a MHH programme for schoolgirls. The tendency toward single use pads, which have dominated the product regulatory market, places additional burden on WASH facilities, around consistent supply, cost, issues about seeing blood which is culturally unacceptable, and waste disposal. Menstrual cup use, including among FSW would help to alleviate these issues.

The aim of this assessment was to evaluate WASH facilities where sex work takes place, as deficits may impact participants' ability to manage their menstrual needs. Our parent study aims to evaluate whether menstrual cups impact the vaginal microbiome, BV, and STIs among FSW who rely on sex for economic livelihood. In this sub-study, we found that WASH facilities in places where sex work takes place are commonly unacceptable, and thus reflect on how this can impact on safe cup use. A systematic review and meta-analysis demonstrated that menstrual cups have a good safety profile, with very rare serious adverse events associated with their use (24). Infection with *Staphylococcus aureus* has not been evident in real-life studies, with no difference in prevalence rates across differing menstrual products (43, 44). However, cup contamination due to poor WASH environments could be a consideration. For example in rural schools in western Kenya where WASH facilities were inadequate, (30). *E. coli* found on the cups was associated with initial use of cups, though the rate of *E. coli* recovery decreased among experienced cup users (43).

### 4.1 Limitations

In the nation-wide key population size estimation exercise conducted 2017–2018 (20), there were an estimated 5,151 FSW in Kisumu, with 438 hotspots identified (20). Of these 438 venues, 130 were in Kisumu Central, where our WASH assessment took place. While we cannot determine whether sex work hotspots included in our study are the same as those in the 2017/2018 assessment, our data may represent between 30%−40% of FSW hotspots in Kisumu City. The national findings did not report venue type or frequency by geographic location, so we cannot fully assess generalizability of our assessment. However, the majority of venues assessed in our study were bars (with and without lodging), which is in keeping with the national key population hotspot estimation exercise, where they comprised 75% of hotspots; streets/alleys/highways accounted for 5%, guest house/lodging 4.4%, and brothels (1.6%). Another strength of our study was the use of direct observation, as self-reported measures of sanitation have been shown to have low reliability (45).

This study was conducted within Kisumu City, and may thus over-represent quality of service provision for Kisumu County. For example, WASH facilities within a central business district may have the advantage of being on municipal water supply, and sanitation system within the town infrastructure. While our WASH assessments took place at a range of areas in Kisumu City, we could not assess differences by town location due to limited number of type of venues within an area. Such analyses would be impeded by sparsity and would compromise confidentiality. Of the 183 latrines counted on premises, we were able to assess 151 (82.5%), due to others being “in use” or “unavailable.” While we cannot provide evidence, study co-authors who conducted the assessments suspected that at least some of these “unavailable” latrines were likely of lower standards and proprietors were not wanting to show them. We classified venues as having an acceptable latrine if there was at least one latrine meeting the criteria, but if there were very many users, this may not have been acceptable. We did not ask proprietors about the average numbers of men and women visiting the venues or how this varied at peaks days and times, and future assessments of WASH at sex work venues should attempt to assess this. We also note that assessing the end of the service chain for disposal of waste material was outside the scope and ability of interviewers to ascertain. We recommend that future studies include this neglected component of MHH.

## 5 Conclusions

Good quality WASH interventions go beyond the immediacy of cleaning the body, and are needed to support all menstruator's wellbeing and self-autonomy (15), and reduce violence (14), infections (16, 17), stigma and discrimination (10). WASH facilities currently available for FSW in this urban area of western Kenya are inadequate for their needs, especially in relation to MHH. Research is needed to identify acceptable and cost-effective approaches to sustainably improve WASH facilities in places of sex work.

## Data availability statement

The datasets presented in this study can be found in online repositories. The names of the repository/repositories and accession number(s) can be found below: de-identified WASH data. Final analytic dataset of 47 records. Supplementary Table of WASH Checklist is the modified UN tool that was used and provides dictionary to dataset. University of Illinois at Chicago. Dataset. https://doi.org/10.25417/uic.24224779.v1.

## Author contributions

PP-H: Funding acquisition, Investigation, Methodology, Writing – original draft. EO: Investigation, Project administration, Supervision, Writing – review & editing. CA: Investigation, Project administration, Writing – original draft. GZ: Data curation, Investigation, Methodology, Writing – review & editing. FO: Investigation, Supervision, Writing – review & editing. SM: Conceptualization, Data curation, Formal analysis, Funding acquisition, Investigation, Methodology, Visualization, Writing – review & editing.

## Supplementary material

Supplementary Table 1 WASH checklist.
