# Supplementary material for: Water, sanitation and hygiene at sex work venues to support menstrual needs
Source: Front Public Health. 2024 Feb 28;12:1305601. doi: 10.3389/fpubh.2024.1305601 (PMC10936742; doi:10.3389/fpubh.2024.1305601)
Supplement: Supplementary Table 1 — WASH checklist. [file Data_Sheet_1.PDF]

POWER Health, WASH CHECKLIST v1.23

GENERAL INFORMATION

LOCATION NAME: ________________________ AREA: __________________________________

DATE: |__||__|/|__||__|/|__||__|

STUDY STAFF INITIALS (must be at least 2 staff): |__||__||__| |__||__||__| |__||__||__| |__||__||__|

TYPE OF VENUE: ☐1 Street ☐2 Guest House ☐3 Hotel ☐4 Bar ☐5 Private Home ☐7 Restaurant

[select all that apply] ☐8 Brothel ☐6 Other: _____________________________

CORE WASH

W1. What is the main source of drinking water provided by the venue or location? (check one – most frequently used)

Improved
- ☐ Piped
- ☐ Tube well/Borehole
- ☐ Protected dug well
- ☐ Protected spring
- ☐ Rain water
- ☐ Tanker truck
- ☐ Other:_______

Unimproved
- ☐ Unprotected dug well
- ☐ Unprotected spring
- ☐ Surface water (River/Lake/Canal)
- ☐ No water source

If Street based venue: Is there a public toilet available?

☐ NA, not street based ☐ Yes, public toilet available ☐ No, no public toilet available

If street-based venue, and public toilet is available, please follow the checklist for this public toilet.

Please write the approximate location of this public toilet:

____

W2. Is drinking water from the main source currently available at the venue or location?

☐ Yes ☐ No

S1. Type of toilets/latrines (select one – most common):

Improved
- ☐ Flush/Pour-flush to sewer
- ☐ Flush/Pour-flush to tank or pit
- ☐ Flush/Pour-flush to open drain
- ☐ Pit latrine with slab/covered

Unimproved
- ☐ Pit latrine without slab/open
- ☐ Bucket
- ☐ Hanging toilet/latrine
- ☐ None

S2 & S3 (alt) How many toilets/latrines are at the venue (insert number)?

	Women's only toilets	***Men's only toilets***	***Common use toilets***
Total number			
Number that are currently usable (available, functional, private			

For guest houses, insert number of self-contained toilets within the rooms

CCG1. Is there a privacy wall separating women's latrines from men's latrines?

☐ Latrines are completely separate
☐ Latrines are joined, and there is a privacy wall separating women's and men's latrines
☐ Latrines are joined and there is no privacy wall separating women's and men's latrines
☐ Common use latrines only

For each toilet/latrine up to 10 toilets/latrines, please record yes/no for each.

Latrine Number	Stable	Holes in Wall	Strong smell of urine or feces	Urine or Feces on floor	Roof	Door	Door locks inside	Functional lighting
1								
2								
3								
4								
5								
6								
7								
8								
9								
10								

*For each toilet, indicate if “not available/in use”.

Please take photo, if allowed, of a latrine that is representative of the conditions.

XW1. How many drinking water points (e.g. taps) are at the venue?
☐ Write Number _______________

XW6a. Does the venue do anything to the water from the main source to make it safe to drink?
☐ Yes ☐ No

XW6b. If yes, what treatment method is used?
☐ Filtration
☐ Boiling
☐ Chlorination
☐ Solar/SODIS
☐ Ultraviolet
☐ Other ____________________

H1. Are there handwashing facilities at the venue?
☐ Yes ☐ No

H2. Are soap and water **currently** available at the handwashing facilities?
☐ Yes, both water and soap
☐ Water only
☐ Soap only
☐ Neither water or soap

XH1. Where are handwashing facilities with water and soap located at the venue? *(mark all that apply)*
☐ Toilets
☐ Food preparation area
☐ Food consumption area
☐ Yard
☐ Rooms
☐ Other ____________________

XH2. How many handwashing facilities with water and soap are located at the venue? *(insert number of taps)*
☐ Total number of taps _________ ☐ Number with soap & water _________

XS1. Are water and soap available in a private space for women to manage menstrual hygiene?
☐ Yes, water and soap ☐ Water, but no soap ☐ No water or soap

CCG2. Separate from the toilets/latrines, is there a private place where women can change or clean themselves?
☐ Yes ☐ No

XS2. Are there disposal mechanisms for menstrual hygiene waste at the venue?
☐ Yes ☐ No

CCG3. How are sanitary pads disposed of at the venue?

☐ Thrown in pit latrine ☐ Placed in a bin and collected by municipal services ☐ Rubbish pit and burned on premises ☐ Rubbish pit and buried on premises ☐ Women take them with them ☐ Don't Know

☐ Other ________________________________

XS3. Are there covered bins for disposal of menstrual hygiene materials in women's toilets?
☐ Yes ☐ No

XS6. Is there at least one usable toilet/latrine that is accessible to women only at the venue? ☐ Yes ☐ No

XS6a. Is there at least one usable toilet/latrine that is accessible to women that men can also use? ☐ Yes ☐ No

XS7. Where are the women's toilets located? [check all that apply]
☐ Within building
☐ Outside building, but on premises
☐ Off premises

XS7B. If Off Premises, What is the distance from the premise? Within 1 minute walk, within 3 minutes walk, more than 3 minutes walk.

XS8. When are women permitted to use the toilets/latrines? (applies to women's only toilets *or* common use toilets)
☐ At all times during the day
☐ During specific times during the day
☐ There are no toilets available for use at the venue

XS4. How many times per week are the toilets cleaned?
☐ At least once per day
☐ 2-4 times per week
☐ Once per week
☐ Less than once per week

XS5. In general, how clean are the toilets?
☐ Clean
☐ Somewhat clean
☐ Not clean
☐ Not assessed (latrines unavailable, in use, or none on site)

XS10. Are latrines or septic tanks emptied (or latrines safely covered) when they fill up?
☐ Yes ☐ No ☐ Unknown

XH3. How is solid waste (garbage) from the venue disposed of?
☐ Private waste collection system
☐ Burned on premises
☐ Buried and covered on premises
☐ Openly dumped on premises

Notes: ____ [Open text]
